# Identification of Floral Scent in Chrysanthemum Cultivars and Wild Relatives by Gas Chromatography-Mass Spectrometry

**DOI:** 10.3390/molecules20045346

**Published:** 2015-03-25

**Authors:** Hainan Sun, Ting Zhang, Qingqing Fan, Xiangyu Qi, Fei Zhang, Weimin Fang, Jiafu Jiang, Fadi Chen, Sumei Chen

**Affiliations:** 1 College of Horticulture, Nanjing Agricultural University, Nanjing 210095, China; E-Mails: sunhainan1989@hotmail.com (H.S.); 2014204028@njau.edu.cn (T.Z.); 2013104100@njau.edu.cn (Q.F.); qixiangyu1005@163.com (X.Q.); zhangfei@njau.edu.cn (F.Z.); fangwm@njau.edu.cn (W.F.); jiangjiafu@njau.edu.cn (J.J.); 2 Jiangsu Province Engineering Lab for Modern Facility Agriculture Technology & Equipment, Nanjing 210095, China

**Keywords:** *Chrysanthemum morifolium*, floral scent, GC-MS, SPME, volatile

## Abstract

The objective of this study was to identify the major volatile compounds and their relative concentrations in flowers of different chrysanthemum cultivars and their wild relatives. The volatile organic components of fresh flowers were analyzed using a headspace solid-phase microextraction coupled with gas chromatography-mass spectrometry. In total, 193 volatile organic components were detected; the major scent components were monoterpenoids and oxygenated monoterpenoids, which accounted for 68.59%–99.93% of the total volatiles in all tested materials except for *Chrysanthemum indicum* collected from Huangshan, in which they accounted for only 37.45% of total volatiles. The major volatile compounds were camphor, α-pinene, chrysanthenone, safranal, myrcene, eucalyptol, 2,4,5,6,7,7ab-hexahydro-1*H*-indene, verbenone, β-phellandrene and camphene. In a hierarchical cluster analysis, 39 accessions of *Chrysanthemum* and its relatives formed six clusters based on their floral volatile compounds. In a principal component analysis, only spider type flowers were located closely on the score plot. The results of this study provide a basis for breeding chrysanthemum cultivars which desirable floral scents.

## 1. Introduction

In many angiosperms, floral volatiles are an important factor in attracting pollinating insects [1]. Floral scents have played important roles in the early evolution of flowers, and most are considered to be pleasant aromas by humans [2,3,4]. There is a never-ending need to characterize and synthesize new aroma compounds [5]. Therefore, over the past decade, there have been many studies on floral scents, their pattern differences among various flowers, and the biosynthesis, emission, regulation and ecological impact of emitted floral volatiles [1,6].

*Chrysanthemum* (*Chrysanthemum morifolium*, tribe Anthemideae, family Asteraceae) is one of 10 well-known traditional Chinese flowers [7]. Together with the rose, it is one of the four most popular cut-flowers in the world. *Chrysanthemum* species account for 30% of world cut flower production [8]. Chrysanthemums are also widely used as a health food and as a Traditional Chinese Medicine (TCM) [9,10]. Early studies on the aroma compounds of *Chrysanthemum* and its wild relatives were largely based on essential oils, and focused on their antioxidant activity and antifungal activity [11,12,13]. The odor compounds in *Chrysanthemum* flowers include β-pinene, eucalyptol, camphor, borneol and bornyl acetate [14,15]. In recent years, there have been some studies on the floral volatile compounds in *Chrysanthemum* and its wild relatives [16,17,18,19]. However, *Chrysanthemum* flower volatiles need to be investigated using more accessions.

Solid-phase microextraction (SPME) is a new type of sample pretreatment technology that allows the rapid and simple extraction of small amounts of volatile compounds. This technique has high reproducibility under the same test conditions and is suitable for floral scent analysis [20]. In this investigation, we performed headspace solid-phase microextraction (HS-SPME) followed by gas chromatography-mass spectrometry (GC-MS) to analyze the floral scent volatiles from 29 chrysanthemum cultivars and five *Chrysanthemum* relatives (a total of 10 accessions) using fresh flowers as plant material. We aimed to identify the cultivars with desirable floral scent by comparing the odor volatiles compositions between different cultivars. Cultivars with desirable scents are promising parents for chrysanthemum breeding.

## 2. Results and Discussion

### 2.1. Flower Scent Volatiles Extraction

The collection of volatile compounds is very important for floral scent analysis. Many methods have been used to collect volatile compounds, including steam distillation (SD), purge and trap, simultaneous distillation extraction (SDE), supercritical-fluid extraction (SFE) and HS-SPME [11,17]. Unfortunately, the aroma of extracted oils rarely represents the delicate natural aroma of blossoms because of thermal artifacts produced during the steam distillation process (which typically occurs at 60–70 °C) [21]. Previous studies on the floral scent of *Chrysanthemum* have analyzed the essential oil, which does not fully represent the delicate natural aroma [11,17]. In this study, we analyzed floral volatiles from chrysanthemum and its wild relatives by extracting compounds from fresh flowers at room temperature by HS-SPME. This method is favorable for floral scent analysis because it minimizes high temperature artifacts [22]. The major compounds in all accessions were monoterpenes and oxygenated monoterpenes, and the minor compounds were sesquiterpenes (Supplementary Table S1). This may be because monoterpenes have a lower boiling point than sesquiterpenes. HS-SPME has been used to analyze volatile compounds emitted from fresh flowers of *Syringa oblata* [23] and tree peony [24]. Thus, collecting fresh flower volatile compounds by HS-SPME coupled with GC-MS represents a sensitive, rapid and effective analytical method for investigating floral aroma compounds in chrysanthemum and its relatives. To our knowledge, this is the first comprehensive study on the aroma of fresh flowers of chrysanthemum cultivars and its wild accessions.

### 2.2. Optimization of HS-SPME Parameters

The scent of *Chrysanthemum* flower is derived from a complex mixture of volatile organic compounds with different polarities, volatilities, functional groups, molecular masses, and concentrations [17]. HS-SPME is an equilibrium method that must be optimized to recover volatiles efficiently and to produce high-quality results. Several organic coatings such as PDMS, DVB and CAR are alternative choices [25]. Temperature also affects the collection of volatiles; as the temperature increases, the recovery of volatile compounds improves, because heat helps to release compounds from the solid sample into the headspace and facilitates the SPME process [26]. In the present study, we obtained fiber equilibrium at room temperature. Thus the floral aroma compounds were extracted under almost “natural” conditions. The other important aspects of SPME sampling are fiber exposure time, amount of sample, handle graduation, and desorption temperature; all of these factors affect the binding of the analyte to the finite sites available on the fiber coating [27].

Previous studies have shown that HS-SPME is a simple, rapid, and efficient technique for quantitative determination of the volatile compounds in *Chrysanthemum* flowers [28], coconut water [27], *Syringa oblata* [23] and grapes [29]. However, the optimum extraction conditions vary according to the plant species. We designed an orthogonal test L_16_ (4^4^) to determine the optimum extraction conditions for *Chrysanthemum*. The importance of the four factors, from most important to least, was A (sample weight) > D (desorb temperature) > B (extraction time) > C (handle scale), when peak area was used as the evaluation index (Table 1). Thus, sample weight had the greatest effect on the test results, followed by desorption temperature, extraction time, and then handle scale. We concluded that the optimal parameter combination was A_2_B_2_C_3_D_4_, according to the mean values of each factor. Therefore, the conditions to achieve optimal results were as follows: sample fresh weight 2.0 g; exposure time of SPME fiber to sample headspace, 30 min at room temperature (25 °C); scale of fiber holder, 3 cm; fiber desorption temperature 250 °C.

**Table 1:** Result of L_16_ (4^4^) orthogonal test for optimization of the HS-SPME parameters.

Code	Sample Weight (A)	Extraction Time (B)	Handle Scale (C)	Desorption Temperature (D)	Peak Area
1	1	1	1	1	2.09 × 10^9^
2	1	2	2	2	1.49 × 10^9^
3	1	3	3	3	1.36 × 10^9^
4	1	4	4	4	1.01 × 10^9^
5	2	1	2	4	2.90 × 10^9^
6	2	2	1	3	2.55 × 10^9^
7	2	3	4	2	3.22 × 10^9^
8	2	4	3	1	1.54 × 10^9^
9	3	1	3	2	2.01 × 10^9^
10	3	2	4	1	1.27 × 10^9^
11	3	3	1	4	2.09 × 10^9^
12	3	4	2	3	3.31 × 10^9^
13	4	1	4	3	1.34 × 10^9^
14	4	2	3	4	3.95 × 10^9^
15	4	3	2	1	8.50 × 10^8^
16	4	4	1	2	9.70 × 10^8^
Mean value 1	1.49 × 10^9^	2.09 × 10^9^	1.93 × 10^9^	1.44 × 10^9^	
Mean value 2	**2.55 × 10^9^**	**2.32 × 10^9^**	2.14 × 10^9^	1.92 × 10^9^	
Mean value 3	2.17 × 10^9^	1.88 × 10^9^	**2.22 × 10^9^**	2.14 × 10^9^	
Mean value 4	1.78 × 10^9^	1.71 × 10^9^	1.71 × 10^9^	**2.49 × 10^9^**	
Range	1.07 × 10^9^	6.08 × 10^8^	5.05 × 10^8^	1.05 × 10^9^	
Optimization level	A2	B2	C3	D4	

### 2.3. Quantitative Evaluation of Floral Scent Volatile Compounds

The 39 accessions showed substantial differences in the total amount of volatiles. The highest concentrations of volatile compounds were in “Nan nong jin kou” and “Hang bai ju” (67.97 and 64.82 µg g^−1^, respectively), and the lowest were in “Nan nong wu feng che”, *Ajania myriantha*, and *C. indicum* collected from Huangshan (2.97, 1.00, and 2.32 µg g^−1^, respectively).

**Figure 1:**
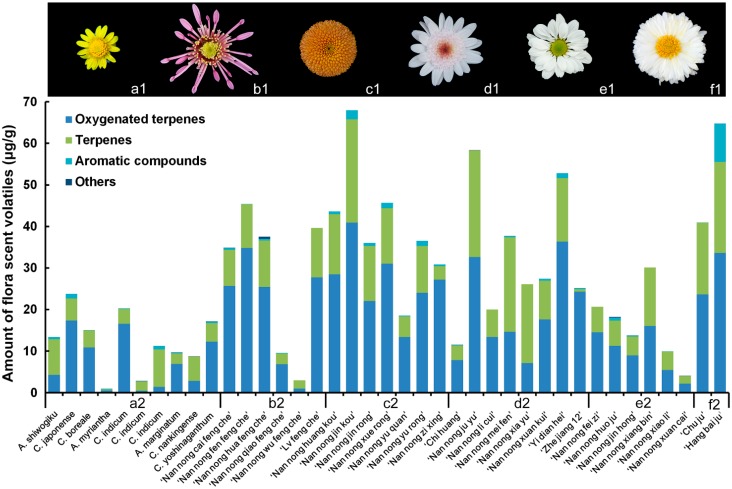
Representative different flower types of *Chrysanthemum* and chemical composition of flower headspace volatiles. (**a**) Wild relatives; (**b**) spider type; (**c**) pompon type; (**d**) anemone type; (**e**) single-flower; (**f**) double-flowers. Individual stacked bars reflect the relative composition of flower volatiles by chemical group.

The chrysanthemum cultivars generally produced larger amounts of volatiles than did their wild relatives (Figure 1). Among the five flower types, pompon and double-flower types produced the largest amounts of volatile compounds, followed by the anemone type, which produced moderate amounts. The single-flower type and wild relatives produced smaller amounts of volatiles than did the other flower types. Different spider type cultivars showed differences in the quantity and quality of floral aroma volatiles (Figure 1). Different accessions of *C. indicum* also showed differences in the amounts of floral scent volatiles. Two cultivars of tea chrysanthemum “Hang bai ju” and “Chu ju”, produced large amounts of floral volatile compounds (Figure 1f2).

### 2.4. Volatile Compounds of Chrysanthemum and Its Wild Relatives

Plants produce diverse secondary metabolites, produce and store a wide range of volatile organic compounds in their flowers. The compound identification was based solely on mass spectrometric data comparison. In total, 193 volatile organic compounds were tentative identified by comparison with known libraries (Table S1); this number is greater than that reported in a previous study [30]. Several of the compounds have been identified previously in *Chrysanthemum* [19]. Different species contained different types and quantities of floral volatile compounds. The number of volatile compounds ranged from 10 (in “Nan nong wu feng che”) to 45 (in “Nan nong jin kou”). Among the 39 accessions, 37 accessions had 10–39 types of volatile compounds; 19 accessions had 20–29 types of volatile compounds; and 2 accessions had more than 40 types of volatile compounds (Table 2).

The 193 volatile compounds were categorized into four groups; terpenes, oxygenated terpenes, aromatic compounds, and others. The terpenes group included 39 monoterpenoids, 27 sesquiterpenes and 17 irregular terpenoids; together, terpenes accounted for 82 of the 193 compounds. All of the oxygenated terpenes were oxygenated monoterpenoids, and included 23 monoterpenoid derivative ketones, 15 monoterpenoid derivative alcohols, 11 monoterpenoid derivative aldehydes and 11 monoterpenoid derivative esters; together, these four types of oxygenated monoterpenoids accounted for 60 of the 193 compounds. There were 27 aromatic compounds and 11 other compounds. These included 13 esters, five alkanes, three alkynes, one olefin, and one nitrogenous compound. Oxygenated terpenes (15.72%–96.53%) and terpenes (2.42%–78.84%) were the major groups of compounds. The contents of these two compounds covered a broad range from 85.55% to 100%, except in *Ajania myriantha*, in which oxygenated terpenes and terpenes accounted for only 41.35% of the total volatile compounds. Oxygenated terpenes and terpenes accounted for at least 95% of the total floral scent volatiles in accessions. Sesquiterpenes accounted for a small percentage (0.00%–4.34%) of total volatiles, except in “Chu ju”, where they accounted for 25.05% of total volatiles. Aromatic compounds also accounted for small proportions (0%–4.89%) of total volatiles in 36 accessions, but *Ajania myriantha*, “Hang bai ju”, and *C. indicum* collected from Huangshan contained higher proportions of aromatic compounds (20.43%, 14.45%, and 11.56%, respectively). The sample of *C. indicum* collected from different locations also showed large differences in the main volatile compounds. Also, differences among the minor components in each chemical group may further distinguish the accessions from each other. All terpenoids and their oxygenated compounds are biosynthesized from the same substrates [31]. The triterpene diols and triols from edible *Chrysanthemum* flowers have been shown to have anti-tumor activity [32,33]. Therefore, “Nan nong jin kou”, “Nan nong ju yu”, “Yi dian hei”, “Nan nong huang kou”, “Nan nong xue rong”, and “Nan nong fen feng che”, which contained higher proportions of triterpene diols and triols, may be candidate cultivars for tea chrysanthemum (Figure 1).

**Table 2:** Quantity of floral aroma volatiles in 39 accessions of *Chrysanthemum*.

Quantity	10–20	20–29	30–39	≥40
Plant accessions	9	19	8	2
Percent	24%	50%	21%	5%

α-Pinene was present in 36 accessions but absent from “Nan nong fen feng che”, “Nan nong ju yu”, and “Zhe jiang 12”. Its concentration ranged from 0.08 µg g^−1^ in “Chi huang” to 10.03 µg g^−1^ in “Nan nong xia yu”, with an average content of 1.24 µg g^−1^ across the 36 accessions. The percentage of α-pinene out of total volatile compounds ranged from 0.56% in “Nan nong yu rong” to 39.14% in *C. indicum* collected from Huangshan. Camphor was present in 35 accessions; its concentration ranged from 0.04 µg g^−1^ in “Nan nong qiao feng che” to 12.44 µg g^−1^ in *C. indicum* collected from Nanjing, and its proportion of total volatile compounds ranged from 0.23% in “Yi dian hei” to 61.36% in *C. indicum* collected from Nanjing. The average content of camphor across 35 accessions was 2.95 µg g^−1^, and its average percentage peak area was 15.75% across all 39 accessions. In addition, camphene, eucalyptol and bornyl acetate were present in at least 30 accessions, while isoborneol, verbenone, bicyclo[3.1.1]hept-2-en-4-ol, 2,6,6-trimethyl-acetate, β-caryophyllene and β-farnesene were present in at least 20 accessions.

### 2.5. Patterns of Floral Scent Volatiles in Accessions of Chrysanthemum and Its Relatives

**Figure 2:**
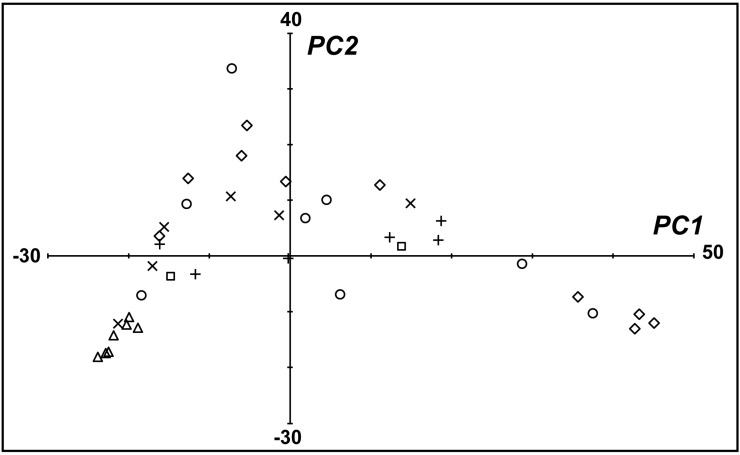
PC1 *vs.* PC2 Eigenvector values of flower volatile values from chrysanthemum cultivars and wild relatives. *Chrysanthemum* wild relatives (◊); spider type (×); pompon type (∆); anemone type (○); single-flower (+); double-flowers (□).

**Figure 3:**
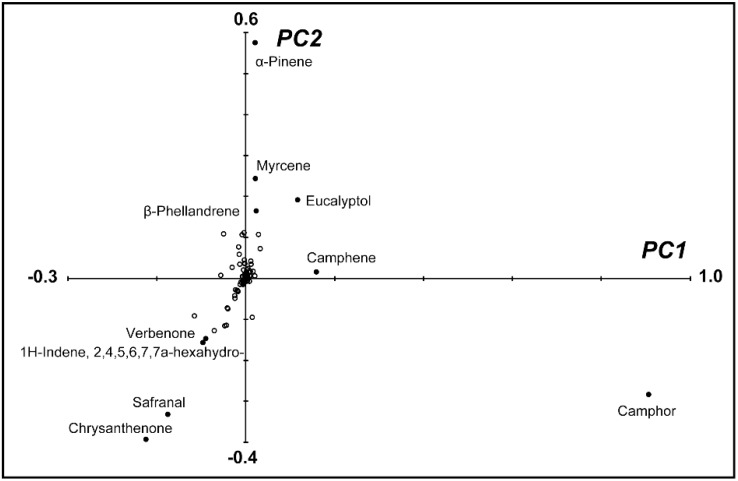
Loading plot of Eigenvector load values of 193 volatile components from PC1 and PC2.

To simplify the multidimensional dataset (193 volatile compounds), we subjected the peak area data to a principle components analysis (PCA) (Table S1). PC1, PC2, and PC3 explained 33%, 11%, and 10%, respectively, of the total variance in the percentages of volatile peak areas. Together, the first three PCs accounted for 54% of the total variance in the percentage of volatile peak areas. Eigenvector values for each sample in the first two dimensions were plotted on a PCA score plot (Figure 2). The pompon type *Chrysanthemum* cultivars clustered together in the PCA based on their scent, while the scents of other flower types and *Chrysanthemum* wild relatives showed wide diversity. We created a 2D loading plot (Figure 3) to further explore the influence of the floral amore volatiles on the differentiation of the 39 accessions. The components that explain more variance in the data have higher loading values, and those explaining less variance or no variance have low or zero loading values, respectively. Ten compounds with the highest loading values are annotated (Figure 3). As follows: Camphor (0.91 in PC1 and −0.28 in PC2; highest loading value for PC1); α-pinene (0.02 in PC1 and 0.58 in PC2; highest loading value for PC2); chrysanthenone (−0.23, −0.39); safranal (−0.18, −0.33); myrcene (0.02, 0.24); eucalyptol (0.12, 0.19); 2,4,5,6,7,7ab-hexahydro-1*H*-indene (−0.10, −0.16); verbenone (−0.09, −0.15); β-phellandrene (0.02, 0.16); and camphene (0.16, 0.01).

**Figure 4:**
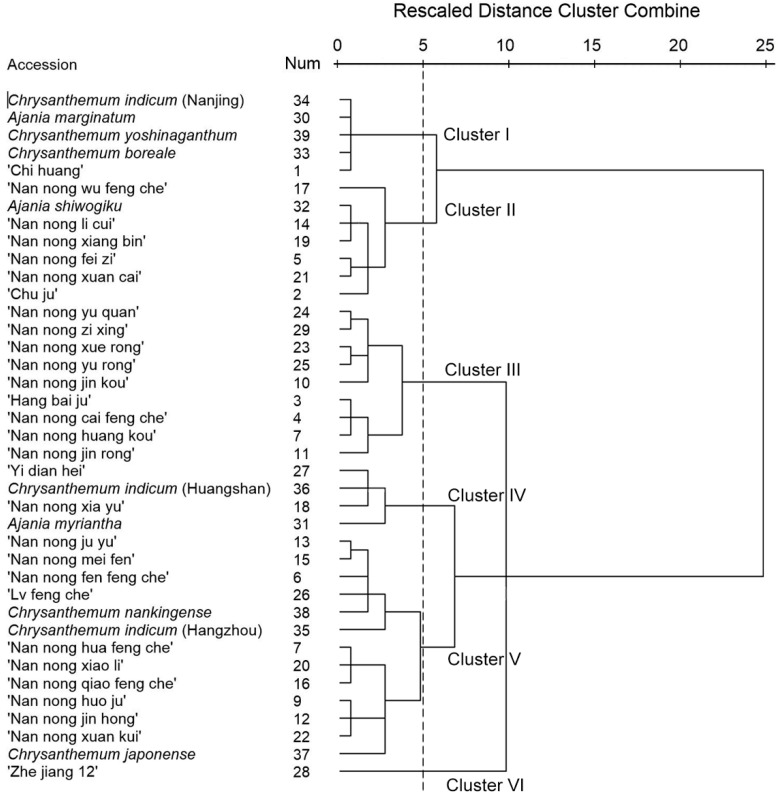
Hierarchical cluster dendrogram of 39 plant materials.

To compare volatile compounds compositions among the 39 accessions, we performed a hierarchical cluster analysis based on the relative contents of the 193 aromatic volatile compounds. We used Ward’s method for between-groups linkage, and the squared Euclidean distance between clusters as a proximity measurement. The 39 accessions formed six clusters in the dendrogram (Figure 4). Five accessions were in cluster I, in which camphor (50.56%–61.36%) and camphene (6.23%–10.39%) were the major components. Seven accessions were in cluster II, in which camphor (24.48%–42.62%) was also a major component, but at lower concentrations than in cluster I, with eucalyptol (0%–30.21%) as the other major component. *Chrysanthemum* are used as TCM for a long time, and its active compounds are eucalyptol and camphor [32]. An analysis of the chemical composition and antimicrobial activity of *C. indicum* essential oils showed that those obtained from processed flowers had a higher percentage of camphor and greater bacteriostatic activity than those obtained from air-dried flowers [11]. Our results suggest that the accessions in clusters I and II are suitable for TCM because of their high camphor content. Cluster III consisted of eight accessions, with α-gurjunene (0%–21.43%) and 3-tert-butylphenol (0%–23.95%) being the major components. Four accessions were in cluster IV, in which α-pinene (1.14%–39.14%) was the major component. There were 13 accessions in cluster V, but there was no consistent major compound among their floral scent volatiles, and they were not grouped based on plant taxonomy or flower shape.

*Chrysanthemum* cultivar “Zhe jiang 12” formed its own cluster, with thujone (65.51%) and α-thujone (19.31%) as the major compounds. Because the compound identification was based solely on mass spectrometric data comparison, stereochemical structure of these major compound were not clearly identified yet. It is necessary to making the stereochemical structure clearly in our ongoing study. These major compounds may also help us to choosing promising cultivars of elegant floral scent parents for chrysanthemum breeding.

## 3. Experimental Section

### 3.1. Plant Materials

Twenty-nine *C. morifolium* cultivars, three *Ajania*, and five wild relatives of *Chrysanthemum* were used in this study (Table 3). All plants were obtained from the Chrysanthemum Germplasm Resource Preservation Centre, Nanjing Agricultural University, China.

**Table 3:** Chrysanthemum cultivars and wild relatives included in this study.

Code	Accessions	Collection Locality
1	*Chrysanthemum morifolium* “Chi huang”	Nanjing, Jiangsu province, China
2	*Chrysanthemum morifolium* “Chu ju”	Nanjing, Jiangsu province, China
3	*Chrysanthemum morifolium* “Hang bai ju”	Nanjing, Jiangsu province, China
4	*Chrysanthemum morifolium* “Nan nong cai feng che”	Nanjing, Jiangsu province, China
5	*Chrysanthemum morifolium* “Nan nong fei zi”	Nanjing, Jiangsu province, China
6	*Chrysanthemum morifolium* “Nan nong fen feng che”	Nanjing, Jiangsu province, China
7	*Chrysanthemum morifolium* “Nan nong hua feng che”	Nanjing, Jiangsu province, China
8	*Chrysanthemum morifolium* “Nan nong huang kou”	Nanjing, Jiangsu province, China
9	*Chrysanthemum morifolium* “Nan nong huo ju”	Nanjing, Jiangsu province, China
10	*Chrysanthemum morifolium* “Nan nong jin kou”	Nanjing, Jiangsu province, China
11	*Chrysanthemum morifolium* “Nan nong jin rong”	Nanjing, Jiangsu province, China
12	*Chrysanthemum morifolium* “Nan nong jin hong”	Nanjing, Jiangsu province, China
13	*Chrysanthemum morifolium* “Nan nong ju yu”	Nanjing, Jiangsu province, China
14	*Chrysanthemum morifolium* “Nan nong li cui”	Nanjing, Jiangsu province, China
15	*Chrysanthemum morifolium* “Nan nong mei fen”	Nanjing, Jiangsu province, China
16	*Chrysanthemum morifolium* “Nan nong qiao feng che”	Nanjing, Jiangsu province, China
17	*Chrysanthemum morifolium* “Nan nong wu feng che”	Nanjing, Jiangsu province, China
18	*Chrysanthemum morifolium* “Nan nong xia yu”	Nanjing, Jiangsu province, China
19	*Chrysanthemum morifolium* “Nan nong xiang bin”	Nanjing, Jiangsu province, China
20	*Chrysanthemum morifolium* “Nan nong xiao li”	Nanjing, Jiangsu province, China
21	*Chrysanthemum morifolium* “Nan nong xuan cai”	Nanjing, Jiangsu province, China
22	*Chrysanthemum morifolium* “Nan nong xuan kui”	Nanjing, Jiangsu province, China
23	*Chrysanthemum morifolium* “Nan nong xue rong”	Nanjing, Jiangsu province, China
24	*Chrysanthemum morifolium* “Nan nong yu quan”	Nanjing, Jiangsu province, China
25	*Chrysanthemum morifolium* “Nan nong yu rong”	Nanjing, Jiangsu province, China
26	*Chrysanthemum morifolium* “Lv fengche”	Nanjing, Jiangsu province, China
27	*Chrysanthemum morifolium* “Yi dian hei”	Nanjing, Jiangsu province, China
28	*Chrysanthemum morifolium* “Zhe jiang 12”	Nanjing, Jiangsu province, China
29	*Chrysanthemum morifolium* “Nan nong zi xing”	Nanjing, Jiangsu province, China
30	*Ajania marginatum*	Tsukuba, Japan
31	*Ajania myriantha*	Jinchuan, Sichuan province, China
32	*Ajania shiwogiku*	Tsukuba, Japan
33	*Chrysanthemum boreale*	Tsukuba, Japan
34	*Chrysanthemum indicum*	Nanjing, Jiangsu province, China
35	*Chrysanthemum indicum*	Hangzhou, Zhejiang province China
36	*Chrysanthemum indicum*	Huangshan, Anhui province, China
37	*Chrysanthemum japonense*	Tokyo, Japan
38	*Chrysanthemum nankingense*	Nanjing, Jiangsu province, China
39	*Chrysanthemum yoshinaganthum*	Tsukuba, Japan

The plants were grown in a greenhouse at 23–28 °C and 80% relative humidity under a natural photoperiod. The flower stems were harvested between 9:00 and 11:00 AM, and immediately placed in deionized water at 25 °C. The stem length was 90 cm, according to the chrysanthemum cut flower industry standard. Five types of chrysanthemum flowers were represented: spider; pompon; anemone; single and double-flower types (Figure 1). Individuals of each cultivar and wild species were asexually propagated by cuttings from the same mother stock plant.

### 3.2. Sample Preparation

To minimize mechanical wounding, fully open intact inflorescences (capitulum) were cut from the stem with a sharp scalpel blade, and ca. 2.0 g fresh weight of intact inflorescences (3–6 inflorescences for cultivars, 15–30 inflorescences for wild species) was weighed and placed in a 40 mL brown glass vial capped with a polytetrafluoroethylene septum with an aluminum cap (Agilent Technologies Inc., Santa Rosa, CA, USA). The experiment was conducted in triplicate.

### 3.3. Optimization of HS-SPME Parameters

A 50/30 µm DVB/CAR/PDMS coated SPME fiber (Supelco Inc., Bellefonte, PA, USA) attached to a manual SPME holder (Supelco Inc.) was used to extract floral volatiles. The sample weight, fiber exposure time, handle scale, and desorption temperature can affect the extraction efficiency. We designed an orthogonal test L_16_ (4^4^) to optimize these four parameters (Table 4) for “Nan nong huo ju”. The vial temperature was 25 °C. Ethyl decanoate (110-38-3, ≥98%, Sigma Aldrich, St Louis, MO, USA) was diluted to 43.25 ng mL^−1^, and 10 µL was added to the glass vial as an internal standard. Peak area was chosen as the evaluation index. Samples were equilibrated for 20 min at room temperature before analysis, and the fiber was conditioned in the GC injection port for 1 h at 250 °C before the first volatile collection, according to the manufacturer’s recommendations. When the extraction of floral volatiles was complete, the fibers were immediately thermally desorbed in the injection port for 5 min. After analysis, the fibers were thermally desorbed in the GC injector to prevent contamination.

**Table 4:** L_16_ (4^4^) orthogonal test to optimized HS-SPME parameters.

Level	Factor			
	Sample Weight (A/g)	Extraction Time (B/min)	Handle Graduation (C/cm)	Desorption Temperature (D/°C)
1	2.5	40	1	220
2	2.0	30	2	230
3	1.5	20	3	240
4	1.0	10	4	250

### 3.4. GC-MS Conditions

Flower volatiles were analyzed using a GC-MS system (7890A-5975C, Agilent Technologies Inc.) equipped with a HP-5MS capillary column (30 m × 0.25 mm, 0.25 µm film thickness, Agilent Technologies Inc.). Helium (99.999%) was used as the carrier gas at a flow-rate of 1 mL·min^−1^ (no split injection). The injection temperature was set to the optimum conditions established in the orthogonal test. The column oven temperature program was as follows:40 °C for 1 min, increasing to 80 °C at a rate of 20 °C·min^−1^, then to 160 °C at 5 °C·min^−1^, then to 270 °C at 20 °C·min^−1^; hold for 1 min. The ion source and quadrupole temperatures were 230 °C and 150 °C, respectively. The ionizing energy was 70 eV and the mass range scanned was 40–500 amu in the full-scan acquisition mode.

### 3.5. Peak Identification

Peaks in the chromatogram were tentative identified based on comparisons with those in the NIST08 (National Institute of Standards and Technology, Gaithersburg, MD, USA) mass spectral library. Each volatile compound was quantified based on comparison of its peak area with that of the internal standard, which was added at a known concentration. The following formula was used to calculate the concentration of each component: content of aroma component (ng g^−1^) = peak area of each component/peak area of internal standard × concentration of internal standard (ng μL^−1^) × volume of internal standard (µL)/fresh weight of sample (g).

### 3.6. Statistical Analyses

The hierarchical cluster analysis, which was based on the relative contents of all aromatic compounds emitted from the 39 accessions, was conducted using SPSS 17.0 (SPSS Inc., Chicago, IL, USA). Ward’s method was used to obtain consistent cluster results, and the squared Euclidean distance between clusters was selected as the proximity measurement [24]. The dataset was subjected to a PCA using Unscrambler 9.7 (Camo Inc. Woodbridge, NJ, USA).

## 4. Conclusions

We determined that the collection of fresh flower volatile compounds by HS-SPME coupled with GC-MS represents a sensitive, rapid and effective method for analyzing *Chrysanthemum* flower aroma compounds. In addition, we optimized the HS-SPME parameters for extraction and analysis of floral aroma volatiles. The 39 accessions showed substantial differences in the total amount of volatiles. The chrysanthemum cultivars generally produced larger amount of volatile compounds than did their wild relatives. Among the five flower morphotypes, only the pompon type cultivars had a characteristic scent. The hierarchical cluster analysis grouped the 39 accessions into six different clusters.

## Supplementary Materials

Supplementary materials can be accessed at: http://www.mdpi.com/1420-3049/20/04/5346/s1.
